# Comparison of Honey & Povidone Iodine dressings in Post-Cesarean Surgical Site Wound Infection Healing

**DOI:** 10.12669/pjms.39.6.7499

**Published:** 2023

**Authors:** Erum Majid, Sadia Pathan, Bader Faiyaz Zuberi, Memoona Rehman, Shoaib Malik

**Affiliations:** 1 Erum Majid, Department of Obstetrics & Gynaecology, JPMC, Karachi, Pakistan; 2 Sadia Pathan, Department of Obstetrics & Gynaecology, JPMC, Karachi, Pakistan; 3 Bader Faiyaz Zuberi, OMI Hospital, Karachi, Pakistan; 4 Memoona Rehman, Department of Obstetrics & Gynaecology, JPMC, Karachi, Pakistan; 5 Shoaib Malik, Anesthesia Department, JPMC, Karachi, Pakistan

**Keywords:** Asepsis score, Honey, Surgical site wound infection, Cesarean section

## Abstract

**Objective:**

To compare the healing of post-cesarean infected wounds by a combination of honey and povidone iodine & povidone-iodine alone dressings using ASEPSIS Score.

**Method:**

This prospective randomised cohort study was carried out at at OBGYN Department Jinnah Postgraduate Medical Center from 1^st^ August 2022 till 31^st^ December 2022. Patients were randomly allocated into groups A and B. Group-A was dressed with honey povidone-iodine, while Group-B only had povidone-iodine. The ASEPSIS score was calculated in both groups on day fifth, 7^th^, 9th, and 10^th^ day. Patients were followed till full recovery of the wound.

**Results:**

A total of 70 women were included and equally allocated into two groups A & B (35 each). On day five mean ASEPSIS score in Group-A was 36.14 and that in Group- B was 37.74. No significant difference in scores were noted on day five [t (68) = -.753, *p* = .454] & day seven Group-A 28.63 vs Group-B 32.11 [t (68) = -1.302, *p* = .197]. Significant improvements in ASEPSIS scores were noted on days ninth & tenth. On day nine ASEPSIS score of Group-A was 21.54 and that of Group-B was 27.14 [t (68) = -2.056, *p* = .044]. On day tenth the mean ASEPSIS score of Group-A was 18.26, while that of Group-B was 23.86 [t (68) = -2.021, *p* = .047]. The mean time required for the wound to heal in Group-A and Group-B was eighteen & twenty-one days respectively.

**Conclusion:**

Significant improvements in ASEPSIS score occurred with the use of honey with povidone-iodine combination.

## INTRODUCTION

The increasing rate of cesarean section has made the rate of Surgical Site Infection (SSI) more devastating with the progress of time. At Kano, Nigeria SSI after cesarean section is reported to be 9.1%. SSI in abdominal surgery at tertiary care hospital in Karachi, Pakistan was reported to be 7.3%.^1^ Robson criteria have been used by WHO to reduce cesarean section but with the increasing population, the rise in the rate of cesarean section is becoming higher over time.^2^-^5^ In JPMC the rate of cesarean section was 36.5% of all live birth in the year 2021.^4^ Similar frequencies of the cesarean section rate of 30% is reported from all hospital births in Australia and New Zealand.^6^,^7^

In this new era, the usage of antibiotics and povidone-iodine was very effective initially but now seems to have limited effect, due to antibiotic resistance developed with frequent use.

SSI related to cesarean section may be due to host-related, pregnancy-related or procedure-related risk factors.^8^ Host-related and pregnancy conditions include Poverty, illiteracy, lack of hygiene, obesity, and medical disorders such as diabetes, hypertension anemia etc. Increasing cesarean section has massively increased the maternal morbidity associated with it.^4^ Infected wound increases the financial burden, hospital stay, and postnatal depression in women, converting their happiness of a baby into a quagmire.

There is a full surah in the Quran emphasizing the importance of honey (Surah Nahl, verse No 68-69).^9^ Honey has been used since 4000 years ago as an anti-inflammatory agent and the Holy Quran describes it to have healing properties.^10^ Honey has gained popularity as it reduces edema, pain, and exudation and improves circulation by increasing angiogenesis. The biochemistry of honey shows that it contains vitamins A, B-complex, D, E, and K, beta-carotene, minerals, and many different enzymes. Honey induces the production of Interleukin 1, 4, and tumor-necrotizing factors by activating WBCs. It has shown good results in multiple sites of wound infection like trauma, burns, malignancy, leprosy, diabetic ulcers, boils, scratches, leg ulcers, gastric ulcers, fistulas, amputation, burst abdominal wounds, septic and surgical wounds, cracked nipples, and wounds in the abdominal wall, but its use in the cesarean section has now recently gained popularity.^10^,^11^

Honey has been observed to have an antimicrobial effect on sixty different organisms along with antifungal activity.^12^ Usually, Antibiotics target a specific site within an infective agent while honey has inhibitory effects that act multi-directionally.^13^ Honey is effective against the most common organism causing wound infection, i.e., Staphylococcus aureus and Methicillin-resistant S. Aureus (MRSA).^14^ The rationale of the study was to compare the effects of honey and povidone-iodine combination with povidone-iodine alone, considering the antiseptic use of honey, its cost-effectiveness, and ease of accessibility.

## METHOD

This prospective cohort study was conducted in the OBGYN Department of a tertiary care hospital of Karachi - JPMC from 1^st^ August 2022 till 31^st^ December 2022**.**

### Ethical approval:

Ethical approval was obtained from Institutional review board of Jinnah Postgraduate Medical Centre under application number: F.2-81/2022-GENL/213/JPMC dated 27-07-2022.

### Sample Size:

The sample size was calculated using reported healing rates of 61.3% in the honey group vs 16.1% in the non-honey group on the following parameters:^15^ the Sample size was calculated for normal approximation alternative hypothesis using a two-sided t-test. Power was kept at 0.95 & alpha at 0.05. Group allocation was assumed to be equal (N1 = N2). Proportions P1 (Group-1 Proportion|H1): 0.613 P2 (Group-2 Proportion): 0.161. Sample size was calculated as N1 = 28, N2 = 28, N = 56. Assuming the dropout rate of 20% the dropout inflated sample size was N1 = 35, N2 = 35, and N = 70. Sample size calculations were done using PASS software.

### Inclusion Criteria:

All post-caesarean patients with wound infection between the age group 18 to 35 years who did not have any risk factors were included after informed consent using convenience consecutive sampling technique.

### Exclusion Criteria:

Subjects with co-morbid such as diabetes, chrioamnionits, preeclampsia, autoimmune disorder, or any other medical condition which delays the healing of the wound were excluded.

### Methods:

The honey used was marketed by Hamdard Shifa Khana and Marhaba available in a tube for easy application on the gauze piece. Dressing was done twice daily in a badly infected wound initially and later once a day when the wound got healthier. Pussy flakes were removed with curettage followed by pouring normal saline. The wound was dried and honey was applied on a 4 x 4 inch gauze piece in the form of thin strip which was kept in the wound gap. Another gauze was applied on the skin followed by sticking plaster.

### Study Instrument:

For the assessment of wound infection, the ASEPSIS scoring method was employed as it was feasible and reproducible. A score of more than 20 was considered sensitive. Wounds were assessed on the day of infection and then on the 5^th^, 7^th^, 9^th,^ and 10^th^ days in both the study groups. The ASEPSIS wound scoring method was created by Wilson AP et al^16^ in 1986 and is used internationally to assess surgical site wound infection. ASEPSIS is an acronym for Additional treatment, Serous discharge, Erythema, Purulent exudate, Separation of deep tissues, Isolation of bacteria, and Stay as an inpatient prolonged over 14 days. It was designed for cardiac patients and had allocated points for each of the Acronyms. Additional points are awarded for antibiotic treatment, drainage of pus under local anesthesia, debridement of the wound under general anesthesia, isolation of bacteria, and stay as an inpatient prolonged over 14 days. Scores are grouped into five categories: satisfactory healing (0-10), disturbance of healing (11-20), minor SSI (21-30), moderate SSI (31-40), and severe SSI (>40). The original ASEPSIS score is meant to evaluate the surgical site for infections from day five to 14 postoperatively, its score allocation is detailed in Table-I.^16^

**Table-I:** ASEPSIS score criteria.

Criteria	Description	Points
Additional treatment	Antibiotics	10
	Drainage of puss	5
	Debridement of wound (General anesthesia)	5
Serous discharge	Daily	0-5
Erythema	Daily	0-5
Purulent exudates	Daily	0-10
Separation of deep tissues	Daily	0-10
Isolation of bacteria		10
Stay in hospital prolonged for >14 days		5

### The CDC describes three types of SSI:^15^

***Superficial incisional SSI:*** This infection occurs just in the area of the skin where the incision was made.

***Deep incisional SSI:*** This infection occurs beneath the incision area in the muscle and the tissues surrounding the muscles.

***Organ or space SSI:*** This type of infection can be in any area of the body other than the skin, muscle, and surrounding tissue that was involved in the surgery. This includes a body organ or a space between organs.

### Data Collection & Analysis:

Patients meeting inclusion criteria were included after informed written consent. Random allocation into two groups was done using a random number table, every patient, on admission, was inducted by referring to the random table and allocated accordingly. Participants doing dressing were resident year II FCPS trainees. They were explained the procedure and dressings were done for one week under principal investigators’ supervision. Asepsis scores were explained to them in detail and they could ask any time from the principal investigator if any problem was encountered. Group-A patients had dressing with povidone-iodine and honey, while Group B had povidone iodine dressing only. Assessment of wound was done using ASEPSIS Score^16^ on days fifth, seventh, ninth and tenth. Antibiotics were given to all patients according to their culture sensitivity. Comparison of means age and ASEPSIS scores between two groups on corresponding days was done by Student’s t-test. Patients were classified into four categories as defined in the methodology based on their scores. Comparision of the categories between two groups was done using the χ^2^-test. The correlation of various quantitative variables was done using Pearson’s Correlation Test. A *p*-value of ≤.05 was considered significant, while ≤.01 was considered highly significant.

## RESULTS

A total of seventy women were included satisfying the inclusion/exclusion criteria after informed consent and were equally allocated into two groups A & B of 35 women each. The mean age of Group-A was 28.43 ±5.24 years and that of Group B was 29.51 ±5.26 years. No significant difference was present between the present age when assessed by Student’s t-test [t (68) = -.865, *p* = .390]. Details of qualitative variables showed that 35, (50 %) of patients studied grade fifth only. Only one participant had a master’s degree. Poor nutrition is again more common in patients with wound infection 44 (62%) Dressing were removed in 64 (78%) of cases in the first 24 to 48 hours. Time required for wound to completely heal was 16 to 20 days with a mean of 18 days in Group-A. In Group-B the average time for complete wound closure was 21.43 days (27 to 15 days). Average BMI was 27 in Group-B, Group-A had an average BMI of 24. Wounds in both groups had a similar length and breadth (Length 6cm and breadth of 3 cm.)

Means of ASEPSIS scores were compared between two groups by Student’s t-test. Significant differences in score means were present on day 09 and day 10. Details are given in Table-II. Healing was also compared by categories as defined in methodology and compared using χ^2^-test and detailed in Table-III.

**Table-II:** Comparison of Mean ASEPSIS scores between groups on assessment days and significance by Student’s t-test.

	Group-A		Group-B		Sig.

	Mean	SD	Mean	SD	
ASS Day 05	36.14	10.15	37.74	7.43	.454
ASS Day 07	28.63	11.55	32.11	10.85	.197
ASS Day 09	21.54	11.98	27.14	10.77	.044*
ASS Day 10	18.26	12.63	23.86	10.45	.047*

* Significance ≤.05.

**Table-III:** Comparison of categories between groups by χ^2^-test.

Duration	Categories	Group				Sig.

		Group-A		Group-B	

		n	%	n	%
ASS Day 05	Satisfactory Healing	2	5.7%	0^1^	0.0%	.470
	Disturbed Healing	0^1^	0.0%	0^1^	0.0%	
	Minor SSI	5	14.3%	6	17.1%	
	Moderate SSI	16	45.7%	19	54.3%	
	Severe SSI	12	34.3%	10	28.6%	
ASS Day 07	Satisfactory Healing	3	8.6%	2	5.7%	.287
	Disturbed Healing	6	17.1%	1	2.9%	
	Minor SSI	11	31.4%	12	34.3%	
	Moderate SSI	8	22.9%	13	37.1%	
	Severe SSI	7	20.0%	7	20.0%	
ASS Day 09	Satisfactory Healing	9	25.7%	4	11.4%	.016*
	Disturbed Healing	9	25.7%	1	2.9%	
	Minor SSI	9	25.7%	18	51.4%	
	Moderate SSI	6	17.1%	9	25.7%	
	Severe SSI	2	5.7%	3	8.6%	
ASS Day 10	Satisfactory Healing	14	40.0%	5	14.3%	.001*
	Disturbed Healing	12	34.3%	7	20.0%	
	Minor SSI	0^1^	0.0%	13	37.1%	
	Moderate SSI	7	20.0%	8	22.9%	
	Severe SSI	2	5.7%	2	5.7%


1. This category is not used in comparisons because its column proportion is equal to zero or one.

* Significance ≤.05

Significant better healing frequency was observed in Group-A. The correlation of Asepsis score on days five, seven, nine, and ten, age, wound length, wound depth, time required for wound healing, time required for the wound to be healthy, and BMI was done by Pearson Correlation Test. Asepsis scores of day nine and day 10 correlated positively with the Time completed for overall wound healing and the Time for the wound to be healthy. BMI correlated negatively with other variables; details are tabulated in Table-IV. Group-A had two patients who turned out to be multi drug resistant (MRSA). One patient in group was multidrug resistant organism. These patients had a delayed wound healing in Group-A, the wound was full length, so they took on an average 45 days for complete closure, as we don’t re-suture MRSA wounds, they are allowed to heal with secondary healing.

**Table-IV:** Correlation coefficient (R) of variables with each other by Pearson Correlation Test.

	Age	BMI	Wound Length	Wound Depth	Time required for Complete healing	Time for a healthy wound	ASS Day 05	ASS Day 07	ASS Day 09
BMI	.037								
Wound Length	-.036	-.034							
Wound Depth	-.078	-.025	.436**						
Time for complete wound healing	.364**	-.208	.207	.235					
Time required for a healthy wound	.240*	-.046	.263*	.389**	.750**				
ASS Day 5	.205	-.178	.070	.202	.408**	.431**			
ASS Day 7	.073	-.088	.122	.091	.379**	.408**	.497**		
ASS Day 9	.073	-.074	.031	.078	.449**	.415**	.416**	.742**	
ASS Day 10	.150	-.148	.142	.011	.492**	.431**	.549**	.806**	.760**

* Significance ≤.05;

** Significance ≤.01.

## DISCUSSION

We observed a faster appearance of granulation tissue after the application of honey and grossly the wound looked red, which was similar to many studies Honey application resulted in a healthier wound within two days (Group-A=Mean asepsis score 36.14 on day five to 28.63 on day seven, while Group-B had an improvement in mean asepsis score from 37.74 on day five to 32.11 on day seven). On day 10 (that is after five days) of dressing Group-A had a mean score of 18.26 which shows minor infection on the contrary Group-B had a score of 23.86. The time required for the wound for complete closure in Group-A was between 16 to 20 days(mean-18), while 14 to 28 days (mean -21) in Group-B, showing an earlier recovery. The wounds were re-sutured after they became healthy, and stitches were removed on the 7^th^ postoperative day. Two patients who had MRSA positive in Group-A that were full length were opened. Honey had a good response in them and proved a good alternative to other expensive dressings. We used simple commercial honey marketed by Hamdard Shifa Khana or Marhaba which gave positive results, keeping in mind their low cost and easy availability. Educating women leads to better recovery and lesser chances of infection is proven by the fact that 41 of the participants of the study were educated till grade 5 only, while 28 had matriculation. Irrigation of the abdomen was beneficial and was found in 22 cases. Uterine exteriorization was done in 37 patients. The dressing was removed in 64 patients in the first 24 to 48 hours and in six patients it was removed in 72 hours. These were the risk factors assessed in the study. By the above findings of the study, we can improve our SSI by irrigation of the abdomen and trying not to exteriorize the uterus while closing it.

The morbidities and resulting financial cost after SSI are the major factors to explore simpler cost-effective agents which are helpful in healing and reducing the time duration required to complete closure of the wound. Knowledge regarding sepsis among doctors is inadequate and needs to be updated.^17^ Internationally Manuka honey dressings are available and are shown to decrease the time of wound healing.^13^ A randomized control trial was conducted by Molan which included 3556 patients, revealing the incredible properties of honey on various types of wounds. A lot of work has been done by the Central for disease control (CDC) to decrease the rate of surgical site wound infection but still, however, we end up having the most fearsome and common complication of wound infection. The study by Okeniyi JA et al showed a 56.5% of the wound were significantly better with honey as compared to Eusol group, while after six days 100% of the wound were clean compared to 65.5% in eusol group.^18^ A case series conducted on 15 posts cesarean scar dehiscence were treated in the same manner as ours with honey applied in the patient’s wound. In all these cases granulation tissue and epithelization were seen in two days.^19^

While comparing this to our data, Studies conducted in ancient times in which sugar was compared with povidone-iodine showed sugar resulted in a lesser requirement for skin graft and early hospital discharge.^11^

There is no clear consensus as to when to remove surgical site dressing as removal in six hours or 24 hours or 48 hours or later on has no advantage over the other.^20^ Placing abdominal binders also give comfort to patients.^21^

The in-vitro antimicrobial analysis of honey shows comparable levels of resistance and sensitivity against S aureus as with trimethoprim. ADMET analysis revealed seven compounds with favorable pharmacokinetic properties comparable to trimethoprim. It was further shown that the bioactive compounds in honey were not inhibitors of the various cytochrome P459 proteins (CYP1A2, CYP2CI9 and CYP2D6) and p-glycoproteins, which further enhanced their bioavailability.^22^ In another study, addition of honey significantly improved the antioxidant activity, resulting in better healing.^23^-^25^ Manuka honey has shown to be effective in treating wounds infected with Escherichia coli, Pseudomonas aeruginosa, Staphylococcus aureus, Extended-Spectrum Beta-Lactamases producing Escherichia coli, Methicillin-resistant Staphylococcus aureus and Candida albicans.^25^

Honey is a broad-spectrum antimicrobial agent which, has long been used in ancient times for treating various infections but due to antibiotic resistance its use is beneficial, supported by antimicrobial stewardship Manuka honey has no resistance till now.^8^ Unlike other antimicrobials honey has a diverse origin derived from different flower nectar comprising high sugar levels, decrease water content, acidity, producing hydrogen peroxide on dilution, and various insect-derived antimicrobials peptides, phytochemicals, and methylglyoxal.

The healing properties of honey are being explored in recent literature and so are emphasized in the Holy Quran. Systematic research with a larger number of patients are required to explore the healing properties of honey to include it in the main treatment of first postoperative dressing in operation theaters as prophylaxis. Honey with no resistance will improve all postoperative recovery of the patients in harmony. This will reduce the Suffering and financial burden making it the need of time.

### Limitations:

It had a low sample size which is its limitation. We used edible honey rather than a medicated one as it was expensive.

## CONCLUSION

The knowledge and science of wound healing is evolving, searching for an ideal compound to treat wounds and prevent infections. Our study results indicate Honey has proven to be more efficacious than povidone iodine only. There were no side effects observed. So, honey proved a good alternative to the more expensive dressings and drugs.

### Authors Contributions:

**EM:** Conceived, designed and prepared the manuscript.

**SP:** Collected the data and entered it in SPSS and is responsible for the integrity of the manuscript.

**BFZ:** Did statistical analysis & editing of the manuscript.

**MR:** Helped in data analyzing and critical analysis of the disease.

**SM:** Helped in designing the study.
